# The Paradoxical Leishmanicidal Effects of Superoxide Dismutase (SOD)-Mimetic Tempol in *Leishmania braziliensis* Infection *in vitro*

**DOI:** 10.3389/fcimb.2019.00237

**Published:** 2019-06-26

**Authors:** Laíse B. Oliveira, Fabiana S. Celes, Claudia N. Paiva, Camila I. de Oliveira

**Affiliations:** ^1^Instituto Gonçalo Moniz-IGM/FIOCRUZ, Salvador, Brazil; ^2^Instituto de Microbiologia, Universidade Federal do Rio de Janeiro, Rio de Janeiro, Brazil; ^3^Instituto de Investigação em Imunologia, São Paulo, Brazil

**Keywords:** *L. braziliensis*, oxidants, anti-oxidants, leishmaniasis, chemotherapy

## Abstract

Leishmaniasis is an infectious disease caused by protozoans of the genus *Leishmania*. The macrophage is the resident cell in which the parasite replicates and it is important to identify new compounds that can aid in parasite elimination since the drugs used to treat leishmaniasis are toxic and present side effects. We have previously shown that treatment of *Leishmania braziliensis*-infected macrophages with DETC (Diethyldithiocarbamate) induces parasite killing, *in vivo*. Thus, the objective of this study was to further evaluate the effect of oxidants and antioxidants in *L. braziliensis-*infected macrophages, following treatment with either oxidizing Hydrogen Peroxide, Menadione, DETC, or antioxidant [NAC (N-Acetyl-Cyteine), Apocynin, and Tempol] compounds. We determined the percentage of infected macrophages and number of amastigotes. Promastigote survival was also evaluated. Both DETC (SOD-inhibitor) and Tempol (SOD-mimetic) decreased the percentage of infected cells and parasite load. Hydrogen peroxide did not interfere with parasite burden, while superoxide-generator Menadione had a reducing effect. On the other hand, NAC (GSH-replenisher) and Apocynin (NADPH-oxidase inhibitor) increased parasite burden. Tempol surfaces as an interesting candidate for the chemotherapy of CL with an IC_50_ of 0.66 ± 0.08 mM and selectivity index of 151. While it remains obscure how a SOD-mimetic may induce leishmanicidal effects, we suggest the possibility of developing Tempol-based topical applications for the treatment of cutaneous leishmaniasis caused by *L. braziliensis*.

## Introduction

Leishmaniasis is zoonotic infection widely distributed from Asia to America which exhibits a high mortality rate. The clinical forms of leishmaniasis depend on the infecting organism and the general state of the host's immune response and are divided in visceral leishmaniasis (VL) and tegumentary leishmaniasis (TL). TL is characterized by cutaneous or mucosal lesions with low lethality, but with high morbidity. CL caused by *Leishmania braziliensis* is distinguished from other leishmaniasis by its chronicity, latency, and tendency to metastasize in the human host (Bittencourt et al., 2003). Brazil along with nine other countries account for 70–75% of the global CL incidence (Alvar et al., 2012). First choice drugs for leishmaniasis chemotherapy are pentavalent antimonials (Sb^v^) [Meglumine Antimoniate (Glucantime®) and Sodium Stibogluconate (Pentostam®) (Croft and Coombs, 2003)] which are significantly toxic and with reported drug resistance (Llanos-Cuentas et al., 2008). Amphotericin B (Annaloro et al., 2009) and Miltefosine (Machado et al., 2010) are also limited with regards to toxicity, cost, and/or time of treatment, reinforcing the need for new chemotherapeutic alternatives.

*Leishmania* promastigotes infect both resident macrophages and monocytes recruited to the infection site. Macrophages are the main host cell, where the parasite differentiates into replicating amastigotes. Upon macrophage activation by IFN-γ, NADPH oxidase generates ${\text{O}}_{2}^{-•}$ through the transfer of electrons from NADPH, coupling them to O_2_. In a phagosome where leishmania parasites reside, ${\text{O}}_{2}^{-•}$ may either undergo SOD degradation to form H_2_O_2_ or be used to generate other ROS, depending on expressed enzymes/cofactors availability and the imbalance between oxidants and antioxidants results in oxidative damage (Sies, 1993). ROS inhibits the growth of *L. braziliensis* amastigotes and contribute to parasite killing (Novais et al., 2014), while NO production alone does not suffice to control infection (Carneiro et al., 2016).

As an evasion strategy, *Leishmania* induces IFN-β production by infected macrophages, which on its turn induces the expression of the enzyme superoxide dismutase (SOD1). The enzyme SOD1 has an antioxidant function: it converts ${\text{O}}_{2}^{-•}$ into molecular oxygen (O_2_) and hydrogen peroxide (H_2_O_2_), the latter degraded by catalase. Survival of *L. amazonensis* and *L. braziliensis* in the host depends on this process (Khouri et al., 2009).

The SOD1-inhibitor diethyldithiocarbamate (DETC) kills intracellular parasites *in vitro* and *in vivo* in a murine model of cutaneous leishmaniasis (Khouri et al., 2010). We have previously shown that DETC can be used as a topical treatment in the cutaneous lesions caused by *L. braziliensis* (Celes et al., 2016), suggesting that manipulation of the redox status during *in vitro* infection with *L. braziliensis* can contribute to the identification of novel therapeutic alternatives. To this purpose, we incubated promastigotes and infected macrophages with Glutahtione replenisher N-acetyl-cysteine (NAC) (Aldini et al., 2018), SOD-mimetic Tempol (Wilcox, 2010) and ${\text{O}}_{2}^{-•}$ -generator menadione (Hassan, 2013). Much to our surprise, we observed that Tempol, a SOD-mimetic, was as effective as DETC (SOD-inhibitor) and menadione (superoxide generator via redox cycling (Criddle et al., 2006) with regards to its ability to reduce macrophage infection by *L. braziliensis*, suggesting novel yet unexplained effects of antioxidants over *Leishmania* infection.

## Materials and Methods

### Ethics Statements

Female BALB/c mice, 6–8 weeks of age, were obtained from IGM/FIOCRUZ animal facility where they were maintained under pathogen-free conditions. All animal work was conducted according to the Guidelines for Animal Experimentation of the Colégio Brasileiro de Experimentação Animal and of the Conselho Nacional de Controle de Experimentação Animal. The local Ethics Committee on Animal Care and Utilization (CEUA) approved all procedures involving animals (CEUA L001/12 IGM/FIOCRUZ).

### Parasites

*Leishmania braziliensis* (MHOM /BR/00/BA788/GFP) were grown in Schneider Insect medium (ThermoFisher Scientific) supplemented with 100 U/mL penicillin, 100 mg/mL streptomycin and 10% inactivated FBS (ThermoFisher Scientific) at 26°C until the stationary phase.

### Infection of Bone Marrow-Derived Macrophages (BMDM) With *L. braziliensis* and Treatment With Oxidants and Anti-oxidants

Bone marrow derived macrophages were obtained as described (Weischenfeldt and Porse, 2008) and were resuspended in DMEM medium (ThermoFisher Scientific) supplemented with 100 U/ml penicillin, 100 ug/ml streptomycin, and 10% inactivated FBS (ThermoFisher Scientific) and seeded at density of 3 × 10^5^ cells per well in 24-well tissue plates. Monolayers received 3 × 10^6^ *L. braziliensis* promastigotes and were incubated at 35 °C in supplemented DMEM medium for 24 h. Infected macrophages were washed to remove non-internalized parasites. Cultures were treated with Diethyldithiocarbamate (DETC) (1 or 2 mM) (Khouri et al., 2010; Celes et al., 2016), Hydrogen Peroxide (100 or 150 μM), N-acetyl cysteine (NAC) (1, 5, or 10 mM), Apocynin (APO) (20 mM) (Paiva et al., 2012), Tempol (4-Hydroxy-TEMPO) (0.5, 1, or 5 mM) (Hahn et al., 1997; Shilo and Tirosh, 2003; Kim et al., 2017) and Menadione (1, 10, or 20 μM) (Mittra et al., 2013), all from SIGMA. Compounds were diluted in DMSO (vehicle). Amphotericin B (0.25 μg/mL, Invitrogen) was used as positive control. After 48 h, cells were extensively washed, fixed, and stained with hematoxylin and eosin (Fischer et al., 2008). The number of infected cells and intracellular amastigotes were counted by optical microscopy in 200 macrophages. Cultures (control and infected macrophages) were performed in quintuplicate. Alternatively, the rate of infection was evaluated by flow cytometry. Briefly, cells were fixed in PBS with 2% paraformaldehyde for 10 min, and kept at 4°C in the dark until acquisition. Data were acquired in a Fortessa flow cytometer (BD Biosciences, USA), for analysis by using FlowJo software (Tree Star, Version 10.2).

### Treatment of *L. braziliensis* Promastigotes With Tempol, DETC, and Menadione

Stationary-phase promastigotes (3 × 10^5^) were cultured in supplemented Schneider in the presence of Tempol (1, 3, or 5 mM), DETC (0.1, 0.5, or 1 mM) and Menadione (2, 5, 10, or 20 μM), all from SIGMA. Promastigotes were cultured in 96-well plates for up to 3 days and the number of viable promastigotes was determined daily using hemocytometer. All assays were performed in quadruplicate and Schneider's medium alone or medium containing vehicle alone were used as a negative control. The half-maximal cytotoxic concentration (CC_50_) and half maximal effective concentration (EC_50_) values of Tempol were determined by a non-linear regression of the concentration-responses curves using GraphPad Software. The selectivity index (SI) was calculated as a ratio between CC_50_/EC_50_ obtained with murine macrophages and intracellular *L. braziliensis* amastigotes, respectively.

### Statistical Analysis

For non-parametric data, analyzes were performed using the Kruskal-Wallis test, followed by the Dunn's multiple comparisons test, for comparisons between three or more groups. For all the analyzes the confidence interval of 95% was established, being the values considered statistically significant when *p*<0.05. Three biological replicates were performed for each experiment. All analyzes were done using GraphPad Prism Software version 5.0. Flow cytometric analyzes were performed using FlowJo software version 10.

## Results and Discussion

Herein, we tested a number of compounds for their ability to modulate the oxidative stress in macrophages infected with *L. braziliensis*. We hypothesized that compounds able to increase ROS induce parasite elimination, building on previous studies showing that such effect can be applied to the development of topical formulations for the treatment of cutaneous leishmaniasis (Celes et al., 2016).

**Figure 1:**
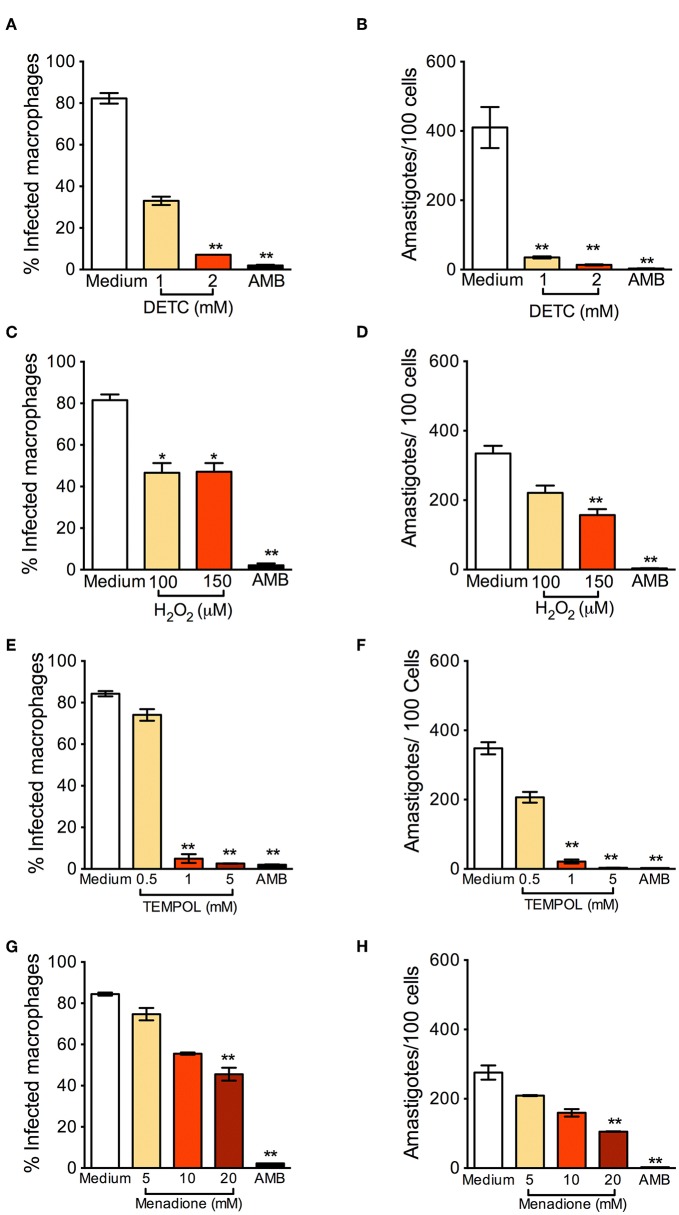
Oxidants reduce *Leishmania braziliensis* infection *in vitro*. Macrophages were infected with *L. braziliensis* for 24 h, and then exposed to different concentrations of DETC **(A,B)**, H_2_O_2_ **(C,D)** for 24 h, Tempol **(E,F)**, and Menadione **(G,H)** for 24 h. Cells were stained with H&E and assessed for the percentage of infection **(A,C,E,F)** and the number of amastigotes per 100 macrophages **(B,D,G,H)** by optical microscopy. Infected macrophages treated with Amphotericin B (AMB) were used as positive controls. Data are shown as mean ± SEM. ^*^p < 0.05; ^**^p < 0.01, ^***^p < 0.001, all comparisons were against negative control (medium).

BMDM were infected with *L. braziliensis* and treated with DETC. DETC significantly reduced the percentage of infected cells (Figure 1A) and the number of intracellular parasites (Figure 1B). DETC (2 mM) showed a leishmanicidal effect similar to that of Amphotericin B, used to treat human leishmaniasis and employed here as a positive control, corroborating our previous findings that the elevation of ${\text{O}}_{2}^{-•}$ levels by DETC-mediated inhibition of SOD1 induces *L. braziliensis* killing (Khouri et al., 2010; Celes et al., 2016). H_2_O_2_ significantly reduced the number of infected cells nor the number of amastigotes (Figures 1C,D, respectively), indicating that the ROS responsible for parasite killing induced by DETC is ${\text{O}}_{2}^{-•}$ itself or another species which uses ${\text{O}}_{2}^{-•}$ as a substrate. These results are in accordance with the killing of *L. braziliensis* amastigotes by EGCG (Inacio et al., 2014), shown to induce the production of superoxide anions, hydrogen peroxide, and other reactive oxygen species (ROS) (Suh et al., 2010), an effect inhibited by catalase-PEG. *L. donovani* was reported to evade oxidative conditions by removing H_2_O_2_ and allowing parasite survival (Channon and Blackwell, 1985). More recently, resistance of *L. donovani*-infected macrophages to H_2_O_2−_ mediated apoptosis was shown to be due to upregulation of thioredoxin and SOCS (Srivastav et al., 2014).

Tempol is an antioxidant able to promote ${\text{O}}_{2}^{-•}$ metabolism at rates similar to SOD and able to permeate membranes freely (Batinic-Haberle et al., 2010). It acts as an ${\text{O}}_{2}^{-•}$ scavenger that crosses cell membranes and therefore can be used to scavenge ${\text{O}}_{2}^{-•}$ in living phagocyte (Gariboldi et al., 1998). We thus expected that Tempol, as an antioxidant molecule, would also favor infection by *L. braziliensis*. Strinkingly, exposure to Tempol significantly reduced the percentage of infected cells (Figure 1E) and the number of amastigotes (Figure 1F), controlling *L. braziliensis* infection, as seen with DETC. Moreover, the combination DETC+Tempol also reduced the percentage of infected cells (Supplemental Figure 1), as seen individually with DETC (Figure 1). Tempol mimics superoxide dismutase activity thus generating hydrogen peroxide and water, destabilizing the oxidation. We can speculate that the presence of Tempol increased H_2_O_2_ levels, interfering with parasite viability. In this case, the concentrations of H_2_O_2_ reached inside the phagosome would need to be significantly higher than those obtained herein following macrophage incubation with 50 μM H_2_O_2_ (Figures 1C,D). Alternatively, Tempol may have off-target leishmanicidal effects superimposed to its anti-oxidant effects. Treatment with Menadione also significantly decreased the percentage of *L. braziliensis*-infected cells (Figure 1G) and the number of intracellular parasites (Figure 1H). We can speculate that, as seen with DETC (Celes et al., 2016), the presence of Menadione may have elevated superoxide levels, leading to parasite elimination.

In *L. infantum*-infected BMDM macrophages, addition of Tempol during phagocytosis increases intracellular infection (Gantt et al., 2001). In *L. amazonensis-*infected mice, SOD-mimetic Tempol exacerbated lesion development and increased parasite load after oral administration. This was associated with reduction of nitric oxide and sequestration of oxidizing molecules (Linares et al., 2008). Differences in the pathogenesis of CL caused by *L. amazonensis* and *L. braziliensis* have been reported regarding the role of neutrophils, for example (Novais et al., 2009; Roma et al., 2016; Carneiro et al., 2018). Therefore, we can speculate that the microbicidal effect of Tempol observed herein, *in vitro*, recapitulate such differences and thus warrant further *in vivo* experiments, especially given Tempol's ability to modulate H_2_O_2_ levels.

We also verified the leishmanicidal effect of oxidants on *L. braziliensis* promastigotes: DETC significantly reduced *L. braziliensis* proliferation at all DETC concentrations tested (Supplemental Figure 2A). As seen with amastigotes (Figures 1E,F), SOD-mimetic Tempol also inhibited the proliferation of *L. braziliensis* promastigotes (Supplemental Figure 2B), similarly to SOD-inhibitor DETC, although we did not observe clear-cut dose-dependent effects. Menadione induced promastigote killing (Supplemental Figure 2C). Lastly, a combination of Tempol+DETC strongly reduced parasite survival as seen with combinations of Menadione + Tempol and Menadione + DETC combinations (Supplemental Figure 2D).

**Figure 2:**
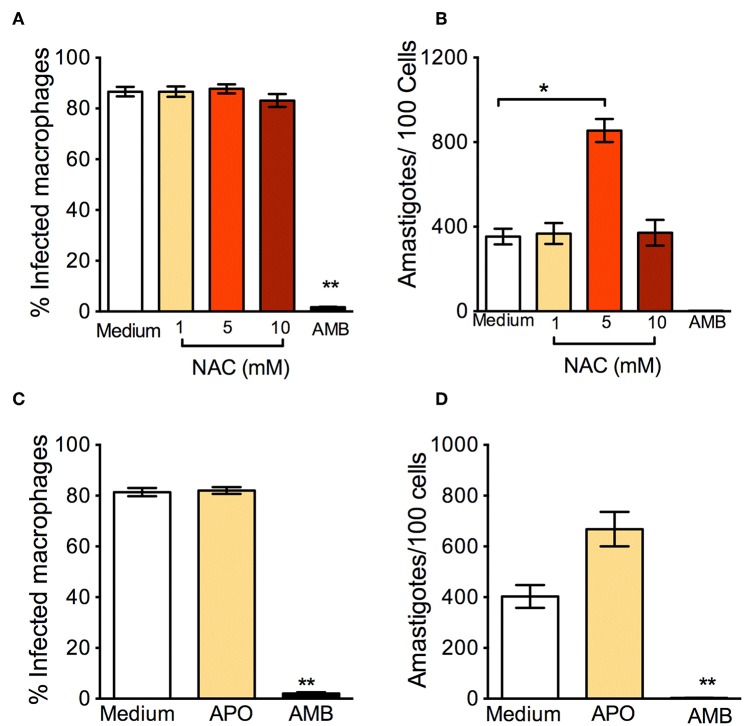
Anti-oxidants enhance *in vitro* infection with *L. braziliensis*. Macrophages were infected with *L. braziliensis* for 24 h, and then exposed to different concentrations of NAC **(A,B)** and Apocycin **(C,D)** for 48 h. Cells were stained with H&E and assessed for **(A,C)** the percentage of infected macrophages and **(B,D)** the number of amastigotes per 100 macrophages by optical microscopy. Infected macrophages treated with Amphotericin B (AMB) were used as positive controls. Data are shown as mean ± SEM. ^*^p < 0.05, ^**^p < 0.01, all comparisons were against negative control (medium).

In parallel to the oxidants, we examined the effect of antioxidants: N-Acetyl-Cysteine (NAC) is a synthetic precursor of intracellular cysteine and glutathione, and its anti-ROS activity results from its ability to directly remove free radicals through the redox potential of thiols and indirectly by increasing levels of glutathione in cells (Sun, 2010). *L. braziliensis*-infected macrophages treated with NAC had a significantly higher percentage of infection (Figure 2A) and a significantly increased parasite load (Figure 2B). Similar results were reported in human monocytes infected with *L. braziliensis* and incubated with NAC (Novais et al., 2014). We believe that these effects are due to the neutralization of ROS, since NAC restores glutathione (GSH) and NAC may protect *L. braziliensis* from oxidative stress just as it does with human red blood cells (Grinberg et al., 2005). Alike NAC, exposure of *L. braziliensis*-infected macrophages to APO did not change the percentage of infected cells (Figure 2C), but induced an increase in the number of amastigotes in cells, indicating that the source of ROS in infected macrophages is indeed NADPH-oxidase respiratory burst (Figure 2D).

Although news studies are needed to understand the mechanisms by which Tempol acts to eliminate *L. braziliensis*, we believe Tempol is an interesting candidate for the chemotherapy of CL. Of note in BMDM, the IC_50_ of Tempol was determined at 0.66 mM ± 0.08 mM, the CC_50_ was calculated as >100 mM and the selectivity index was established at 151. Tempol presents low toxicity and has successfully completed phase I clinical trials to be used topically against tissue damage (Metz et al., 2004). Given that treatment options for CL are currently limited and that the number of refractory cases has increased, Tempol surfaces as a viable alternative for further investigation.

## Ethics Statement

All animal work was conducted according to the Guidelines for Animal Experimentation of the Colégio Brasileiro de Experimentação Animal and of the Conselho Nacional de Controle de Experimentação Animal. The local Ethics Committee on Animal Care and Utilization (CEUA) approved all procedures involving animals (CEUA L001/12 IGM/FIOCRUZ).

## Author Contributions

LO and FC performed experiments. LO, CP, and CO drafted the manuscript. CP contributed reagents.

### Conflict of Interest Statement

The authors declare that the research was conducted in the absence of any commercial or financial relationships that could be construed as a potential conflict of interest.
