# Supplementary material for: The Paradoxical Leishmanicidal Effects of Superoxide Dismutase (SOD)-Mimetic Tempol in Leishmania braziliensis Infection in vitro
Source: Front Cell Infect Microbiol. 2019 Jun 26;9:237. doi: 10.3389/fcimb.2019.00237 (PMC6607107; doi:10.3389/fcimb.2019.00237)
Supplement: Supplementary file 1 [file Data_Sheet_1.PDF]

The paradoxical leishmanicidal effects of Superoxide Dismutase (SOD)-mimetic Tempol in *Leishmania braziliensis* infection *in vitro*

Laise B. Oliveira[1,2], Fabiana S. Celes[1], Claudia N. Paiva[3], Camila I. de Oliveira[1,2,3*]

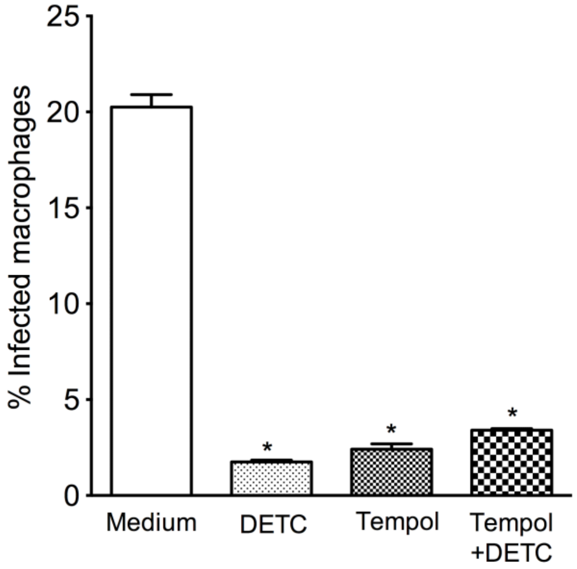

Supplemental Figure 1. DETC and Tempol reduce in vitro infection with *L. braziliensis*. Macrophages were infected with *L. braziliensis* expressing GFP, washed and then and exposed to DETC, Tempol or both. Bar graph depicts the percentage of infected cells as determined by flow cytometry. Data are shown as mean ± SEM.*$p<0.05$, all comparisons were against negative control (medium).

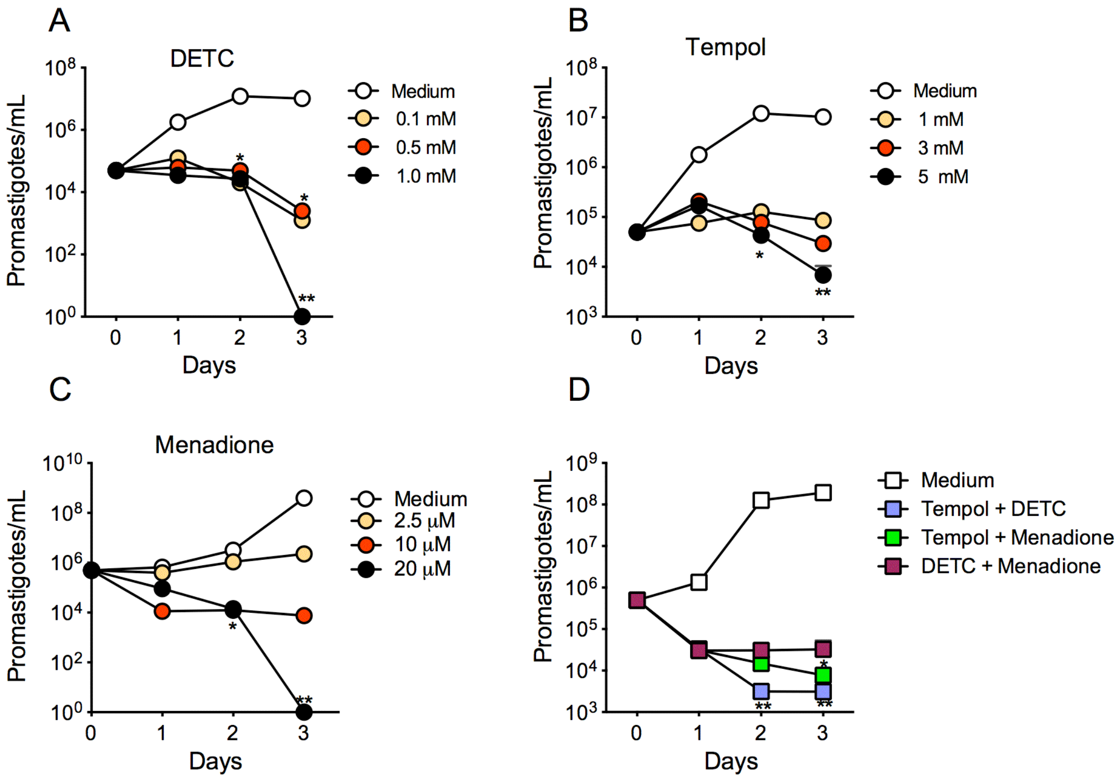

Supplemental Figure 2. Dose-dependent effect of Tempol, DETC and Menadione on *L. braziliensis* promastigotes. *L. braziliensis* promastigotes were grown in medium containing different concentrations of DETC (A), Tempol (B), Menadione (C) or a combination of Tempol + DETC, Tempol+Menadione or DETC+Menadione (D). The number of viable parasites was determined daily by counting in hemocytometer. Data are shown as mean ± SEM. *$p<0.05$; **$p<0.01$, all comparisons were against negative control (medium).
